# Cervical Cancer Epidemiology: Global Incidence, Mortality, Survival, Risk Factors, and Equity in HPV Screening and Vaccination

**DOI:** 10.3390/jcm15031079

**Published:** 2026-01-29

**Authors:** Sara Jouya, Zahra Shahabinia, Afrooz Mazidimoradi, Leila Allahqoli, Hamid Salehiniya, Do-Youn Lee

**Affiliations:** 1 Student Research Committee, Birjand Branch, Islamic Azad University, Birjand 9717853577, Iran; sarahjouya@gmail.com; 2 Geriatric Health Research Center, Birjand University of Medical Sciences, Birjand 9717853577, Iran; shahabiniaz@gmail.com; 3 Iran Health Assistant Department, Shiraz University of Medical Sciences, Shiraz 7193711351, Iran; mazidimoradi.8836@gmail.com; 4 Faculty of Health Sciences, Cyprus International University, Nicosia 99258, Cyprus; lallahqoli@gmail.com; 5 Department of Epidemiology and Biostatistics, School of Health, Social Determinants of Health Research Center, Birjand University of Medical Sciences, Birjand 9717853577, Iran; 6 College of General Education, Kookmin University, Seoul 02707, Republic of Korea

**Keywords:** cervical cancer, human papillomavirus, epidemiology, risk factors, screening, vaccination, health equity

## Abstract

**Background/Objectives:** Cervical cancer remains a major cause of morbidity and mortality among women worldwide, marked by stark geographic and socioeconomic disparities. Preventable via HPV vaccination and screening, progress toward elimination varies widely across and within countries. This narrative review synthesizes the epidemiology, including incidence, mortality, survival, and stage distribution, as well as risk factors and the coverage/equity of HPV screening and vaccination programs. **Methods:** Comprehensive searches were performed in PubMed, Web of Science, Scopus, and Google Scholar (no date restrictions; English only). Included were original epidemiological studies, systematic reviews, meta-analyses, and international reports on burden, risk factors, or prevention indicators. Data were qualitatively synthesized into three themes: epidemiological patterns, risk factors, and screening/prevention programs. **Results:** Persistent high-risk HPV infection causes nearly all cervical cancers, predominantly HPV16/18, with regional variation in other types. Strong co-factors include HIV immunosuppression, early sexual debut, multiple partners, high parity, long-term oral contraceptive use, and smoking. Inequalities in incidence, late diagnosis, and survival are driven by socioeconomic disadvantages, low education, rural residence, and poor health system access. Screening ranges from cytology/VIA to primary HPV testing, but coverage is low and inequitable in high-burden settings. HPV vaccination has expanded yet faces major gaps in low- and middle-income countries. **Conclusions**: Cervical cancer burden concentrates in low-resource and marginalized populations. Global elimination demands accelerated, equitable scale-up of HPV vaccination and screening, alongside health system strengthening and barrier reduction.

## 1. Introduction

Cervical cancer remains one of the leading causes of cancer incidence and mortality among women worldwide, although its burden varies substantially across countries and regions. Global estimates from GLOBOCAN and the Global Burden of Disease (GBD) collaboration indicate that cervical cancer continues to rank among the most common cancers and a major cause of cancer death in many low-income and lower-middle-income countries, whereas in most high-income countries, both incidence and mortality rates are considerably lower, with large differences in age-standardized rates observed across geographic regions [1,2,3]. Within countries, registry-based studies and ecological analyses have consistently demonstrated that women residing in rural areas and socioeconomically disadvantaged or marginalized groups experience higher incidence, are more frequently diagnosed at advanced stages, and have poorer survival outcomes compared with more advantaged populations [4,5,6].

In many high-income countries and some upper-middle-income settings, organized screening programs based on cytology, and more recently HPV-based screening, have led to substantial reductions in both incidence and mortality. In contrast, in numerous low- and middle-income countries where screening coverage remains limited and timely access to treatment is inadequate, rates have either plateaued or, in some cases, increased [7,8,9,10]. Consequently, the World Health Organization (WHO) has declared the global elimination of cervical cancer as an achievable public health goal, provided that high coverage of HPV vaccination, screening, and treatment can be achieved; however, progress toward these targets varies markedly across countries [11,12,13].

Persistent infection with high-risk types of human papillomavirus (hrHPV) is a necessary cause of virtually all cervical cancers. Nevertheless, progression from persistent infection to high-grade precursor lesions and ultimately to invasive cancer depends on a complex interplay of additional behavioral, biological, and structural factors. Virological studies and meta-analyses conducted across multiple countries indicate that HPV16 and HPV18 account for the majority of invasive cancers globally, while types 31, 33, 45, 52, and 58 contribute substantially in certain regions, with notable geographic variation in genotype distribution [14,15,16].

Epidemiological studies and systematic reviews have consistently identified early age at first sexual intercourse, multiple sexual partners, high parity, long-term use of oral contraceptives, tobacco smoking, co-infection with other sexually transmitted infections (STIs), and HIV-associated immunosuppression as important individual-level risk factors [17,18,19,20]. More recent genome-wide association studies (GWAS) and Mendelian randomization analyses further suggest that host genetic factors and genetically proxied behaviors contribute additional, albeit relatively modest, effects [21,22,23,24]. In this synthesis, risk factors were interpreted according to the strength and consistency of evidence, distinguishing necessary causes, strong causal co-factors, and contributory or correlated factors, while GWAS and Mendelian randomization findings were used primarily to support biological plausibility rather than population-level risk prediction. Collectively, these findings indicate that cervical carcinogenesis is a multifactorial process, in which persistent hrHPV infection is necessary but interacts with behavioral, reproductive, hormonal, lifestyle, and host-related factors, and residual confounding between interrelated exposures cannot be fully excluded.

Concurrently, a large body of research has demonstrated that socioeconomic status, educational attainment, place of residence, and health system characteristics substantially influence both the inequalities in cervical cancer burden and the differential implementation and uptake of preventive interventions. Systematic reviews conducted in low- and middle-income settings consistently report low awareness of cervical cancer and markedly reduced participation in screening among women with limited formal education, rural residents, and those facing financial or cultural barriers to health services [25,26,27]. Even in high-income countries, studies show lower screening uptake and later-stage diagnosis (with consequent poorer survival) among women living in deprived neighborhoods, those with disabilities, and uninsured women or those covered only by public insurance schemes [4,28,29,30].

Various screening modalities—from conventional cytology to visual inspection with acetic acid (VIA) and, increasingly, HPV DNA testing—have been implemented at scale in diverse settings. HPV-based strategies generally demonstrate higher sensitivity for detection of high-grade lesions [8,31,32]. Prophylactic HPV vaccination programs implemented early in several countries have achieved substantial reductions in vaccine-type infections and high-grade precursor lesions; nevertheless, many low- and lower-middle-income countries still lack fully funded national programs and continue to rely primarily on opportunistic screening [13,33,34,35].

Despite the extensive research literature, important gaps persist in the comprehensive epidemiological synthesis of cervical cancer. Previous reviews have typically focused on specific aspects—such as global burden, HPV genotype distribution, selected risk factors, or screening test performance—and have often relied heavily on global modeling exercises or clinical trial/program evaluation data [1,8,16,25]. In contrast, narrative syntheses that simultaneously integrate population-based observational studies with global and regional burden estimates, while providing an integrated picture of how infection-related, behavioral, metabolic, genetic, and socioeconomic risk factors intersect with screening and prevention patterns across diverse contexts to produce observed inequalities in disease burden and outcomes, remain relatively scarce [20,27].

Therefore, this narrative epidemiological review aims to provide a comprehensive synthesis of the epidemiology of cervical cancer, including incidence, mortality, survival, risk factors, as well as coverage and equity dimensions of HPV screening and vaccination programs.

## 2. Materials and Methods

This narrative review was conducted through a comprehensive search of the databases PubMed, Web of Science, Scopus, and Google Scholar. No date restrictions were applied; only English-language publications were included. The search strategy combined terms related to “cervical cancer” or “cervix uteri cancer” with keywords such as epidemiology, incidence, mortality, prevalence, survival, risk factor, screening, HPV, and vaccination.

After removal of duplicates, titles and abstracts were screened for relevance, followed by full-text evaluation of potentially eligible records. Inclusion criteria comprised original epidemiological studies, relevant systematic reviews or meta-analyses, and authoritative international reports that provided data on disease burden, risk factors, or indicators of screening/vaccination programs. Non-relevant publications or those lacking sufficient data were excluded.

Data were extracted qualitatively and organized into three main thematic axes: (1) epidemiology (incidence, mortality, survival, stage distribution), (2) risk factors, and (3) screening and prevention programs (coverage, equity, modalities, vaccination).

This review was conducted as a structured narrative synthesis rather than a formal systematic review, given the broad and heterogeneous scope of the evidence. Formal reporting and quality appraisal frameworks were therefore not applied; however, priority was given to population-based studies, large multi-country analyses, systematic reviews, and authoritative international reports.

## 3. Results

### 3.1. Characteristics of Included Studies

In total, 59 articles and reports published between 1999 and 2025 were included in this review. The body of evidence encompassed both international documents providing global/regional burden estimates and policy guidance, as well as original epidemiological studies. The evidence base was highly heterogeneous, drawing from diverse study designs and geographic levels: from comparative global burden estimates [5,36] to national and subnational registry-based analyses focusing primarily on incidence and mortality, with some also reporting stage at diagnosis and survival indicators [4,37,38].

Cross-sectional studies conducted at the community level or within screening programs provided data on HPV infection prevalence, high-grade cervical intraepithelial neoplasia (CIN2+), and screening-related indicators [26,39,40]. Evidence concerning well-established co-factors was strengthened by multi-country pooled case–control analyses among HPV-positive women [17,41] and by large multi-centric European cohort studies examining behavioral and lifestyle factors [42,43].

Additional contributions came from studies on high-risk HPV genotype distribution [14,44], genome-wide association studies (GWAS) and Mendelian randomization analyses of genetic susceptibility [21,22,23,24], and modeling evaluations of HPV-based screening strategies [45].

Overall, the reviewed outcomes included incidence and mortality (with stage distribution and survival when reported), prevalence of HPV infection and precursor lesions, associations between risk factors and disease, and program-level indicators of screening coverage and HPV vaccination.

### 3.2. Epidemiological Patterns of Cervical Cancer

#### 3.2.1. Incidence and Mortality

Recent global burden estimates indicate that cervical cancer continues to represent a major cause of cancer incidence and mortality among women worldwide, with approximately 660,000–670,000 new cases and 300,000–350,000 deaths occurring annually in the early 2020s [3]. In most high-income countries, cervical cancer now constitutes only a small proportion of the overall cancer burden, whereas it remains among the leading causes of cancer death in many low- and middle-income countries, where the large majority of deaths occur [12,46].

Geographic disparities are striking: the highest age-standardized incidence and mortality rates are observed in sub-Saharan Africa, parts of Latin America and the Caribbean, and South and Southeast Asia, while the lowest rates are reported in Western Europe, North America, Australia/New Zealand, and selected West Asian countries [2,36]. Within-country inequalities are also evident, with higher incidence and mortality typically observed among women residing in socioeconomically deprived areas, rural regions, or marginalized urban neighborhoods where screening and vaccination coverage tend to be lower and timely diagnosis/treatment more limited [5,46]. Across recent global analyses, age-standardized incidence and mortality rates in low-SDI settings remain approximately two- to four-fold higher than in high-SDI settings, and screening coverage in rural populations is frequently 30–60% lower than in urban populations, illustrating substantial absolute and relative inequities [4,26,36,46]. While the WHO elimination targets are defined at the national level, these aggregate benchmarks may mask substantial subnational inequities, underscoring the need for equity-sensitive monitoring and targeted implementation strategies [4,11,13,36,46].

#### 3.2.2. Temporal Trends

Global trend analyses from GBD and GLOBOCAN indicate that age-standardized incidence and mortality rates of cervical cancer have generally declined since 1990; however, the pace of progress has been markedly heterogeneous across regions and socioeconomic groups [2,47]. At the same time, absolute numbers of cases and deaths have increased in many settings due to population growth and aging [46].

Rapid declines in age-standardized rates have been documented in many high-income and upper-middle-income countries—particularly in Western and Northern Europe, North America, and Australia/Oceania—consistent with long-standing organized cytology-based screening programs and earlier introduction of HPV vaccination [5,48]. In contrast, analyses stratified by Sociodemographic Index (SDI) and region have reported either increasing or plateauing rates in parts of Eastern Europe, East Asia, and sub-Saharan Africa, particularly in countries classified as medium-high SDI where organized screening remains limited or was only recently initiated [36,49].

Subnational trend analyses further highlight substantial within-country heterogeneity: while some regions have experienced rapid declines, others continue to show persistently high or even rising rates, typically in areas characterized by low or uneven screening coverage [4,37,38,50,51].

#### 3.2.3. Prevalence and Survival

Population- and clinic-based studies consistently demonstrate higher prevalence of hrHPV infection and high-grade precursor lesions (CIN2+) in populations with absent or inadequate screening, particularly among rural and socioeconomically disadvantaged women. For instance, community-based cross-sectional studies conducted in rural areas of India and Bangladesh have reported substantial burdens of HPV infection and CIN2+ among women attending screening, with higher prevalence observed in older age groups and among women with little or no prior screening history [39,40].

Virological analyses across multiple countries confirm that high-risk HPV types are detectable in the vast majority of invasive cervical cancers globally, with HPV16 and HPV18 being the most frequent, although the relative contribution of other carcinogenic types varies substantially by region [14,15,44].

Survival data derived from registry-based and cohort studies uniformly indicate poorer outcomes in low- and middle-income settings, where most women present with advanced-stage disease and access to timely multimodal treatment remains limited [30,52,53]. In high-income countries, survival is generally higher, yet important disparities persist by race/ethnicity, insurance status, and area-level deprivation: Black women and socioeconomically disadvantaged women frequently exhibit lower 5-year survival even after adjustment for stage at diagnosis [28,54].

### 3.3. Risk Factors for Cervical Cancer

#### 3.3.1. Infection-Related Factors (HPV, Other STIs, HIV)

##### High-Risk HPV Infection and Genotype Distribution

Global reports and original studies consistently reaffirm that persistent infection with high-risk human papillomavirus (hrHPV) is the necessary cause of virtually all cervical cancers [3,55]. Virological studies demonstrate that HPV DNA is detectable in the vast majority of invasive tumors, with HPV16 and HPV18 together accounting for the largest proportion of cases worldwide. Other carcinogenic types (31, 33, 45, 52, 58) contribute meaningfully, with their relative importance varying considerably by geographic region [14,15,44] (Table 1).

Population-based screening cohorts report high prevalence of hrHPV among sexually active women, particularly at younger ages and in settings lacking organized screening programs. Prevalence typically declines gradually through middle age, although a smaller second peak is sometimes observed in older age groups [39,56]. Ecological analyses further demonstrate a strong positive correlation between population-level prevalence of hrHPV and cervical cancer incidence, especially in populations with limited screening [5,36].

##### Co-Infection with Other Sexually Transmitted Infections

Relatively few of the original studies included in this review directly and quantitatively assessed the independent role of non-HPV sexually transmitted infections (STIs) as co-factors in cervical carcinogenesis. When mentioned, such infections are usually discussed descriptively or within a broader framework of sexual health rather than as independent exposures. Similarly, global summaries of risk factors emphasize persistent hrHPV infection, sexual behavior patterns, and HIV-related immunosuppression, while providing only limited quantitative data on the specific contribution of other STIs [57,58]. Overall, while biological plausibility exists for co-infections to modify disease progression, their independent epidemiological contribution remains insufficiently quantified in the current body of evidence.

##### HIV Infection and Immunosuppression

Both global reviews and primary studies consistently identify HIV infection and associated immunosuppression as major risk enhancers for cervical cancer. Women living with HIV exhibit higher prevalence and persistence of hrHPV, more frequent multiple-type infections, and substantially increased progression to high-grade lesions [3,55]. Primary studies from sub-Saharan Africa and other high-HIV-prevalence regions report markedly elevated rates of HPV infection, CIN2+, and invasive cancer among HIV-positive women, even at relatively young ages [19,52]. Ecological analyses further reveal strong correlations between regional HIV prevalence and cervical cancer burden, particularly in settings where access to effective antiretroviral therapy (ART) and cervical screening remains limited [2,46].

#### 3.3.2. Sexual and Reproductive Behaviors

##### Age at First Sexual Intercourse and Age at Marriage

Multiple case–control and cross-sectional studies from Asia, Africa, and Europe have reported that earlier age at first sexual intercourse and earlier age at marriage remain associated with increased risk of high-grade precursor lesions and invasive cervical cancer, even after adjustment for other factors [39,56,59]. Women reporting sexual debut in mid-to-late adolescence show higher prevalence of hrHPV and CIN2+ compared with those who initiate sexual activity at older ages—a pattern consistent with increased cumulative opportunity for hrHPV exposure during a potentially more biologically susceptible period [17,60]. Community-based surveys also indicate that early marriage, often involving older male partners, is more prevalent among women with lower educational attainment and in rural areas, highlighting the broader social patterning of early sexual debut and its intersection with limited access to preventive care [26,61].

##### Number of Sexual Partners and Partner Characteristics

Detailed sexual history studies consistently demonstrate that lifetime number of sexual partners is positively associated with risk of cervical cancer and high-grade precursor lesions, with the association being particularly clear in analyses restricted to HPV-positive women. This pattern is thought to reflect increased probability of initial acquisition and subsequent reinfection with hrHPV types [17,60]. Some studies also report elevated risk among women whose male partners have had multiple partners or a history of STIs, consistent with the role of male sexual behavior in transmission [14,56]. Global risk factor summaries continue to identify multiple sexual partners and early, unprotected sexual debut as key behavioral determinants, particularly in settings with low condom use and high STI prevalence [57,58].

##### Reproductive History: Parity, Pregnancies, and Age at First Birth

Reproductive history remains one of the most consistently identified non-viral risk modulators. Pooled analyses and large multi-country case–control studies restricted to HPV-positive women have demonstrated that high parity (number of full-term pregnancies) is associated with significantly increased risk of squamous cell carcinoma, with risk rising in a dose-dependent manner with increasing number of pregnancies [17,62]. Biologically, repeated cervical trauma and epithelial remodeling during multiple pregnancies may facilitate persistent HPV infection and malignant transformation [18,41]. In addition, prolonged exposure to elevated endogenous estrogen and progesterone levels during pregnancy may promote viral persistence and cellular proliferation, thereby increasing carcinogenic susceptibility [18,62,63]. Cohort and case–control studies conducted in diverse settings have reported similar findings: women with three or more full-term pregnancies exhibit a higher risk of CIN3+ and invasive cancer compared with nulliparous or low-parity women. Earlier age at first full-term birth may further amplify risk among HPV-infected women [43,63,64]. Global burden analyses also show that countries with higher average fertility rates and earlier childbearing patterns tend to experience higher cervical cancer incidence, reinforcing the population-level relevance of reproductive patterns [5,46].

#### 3.3.3. Hormonal Contraceptive Use

##### Ever-Use Versus Never-Use of Combined Oral Contraceptives

Several large pooled case–control analyses restricted to HPV-positive women have shown that ever-use of combined oral contraceptives (COCs) is associated with modestly increased risk of invasive cervical cancer and CIN3+ compared with never-use, although effect sizes are generally small [41,62]. This association may partly reflect confounding by correlated sexual and reproductive behaviors, including number of lifetime sexual partners and parity. Although major studies adjusted for key covariates, complete disentanglement of independent effects remains challenging in observational data. Similar patterns have been reported in cohort and case–control studies from Europe, North America, and Asia, with higher risk observed among current or recent users even after adjustment for age, parity, smoking, and sexual behavior [19,63,64]. Global risk factor summaries classify long-term use of oral contraceptives as a probable co-factor in HPV-positive women, while emphasizing that the absolute risk increase remains small relative to the dominant effect of persistent hrHPV infection [57,58].

##### Duration and Patterns of Hormonal Contraceptive Use

Some pooled analyses and individual studies indicate that risk increases with longer duration of COC use, particularly beyond five years of cumulative exposure, with evidence suggesting gradual attenuation of risk following cessation—consistent with a partially reversible effect [41,62]. More recent Mendelian randomization studies using genetically proxied reproductive and hormonal traits have generally supported a modest adverse association with long-term exposure, while also highlighting the potential for residual confounding by fertility patterns and healthcare utilization [24,64]. Data on non-oral hormonal methods (injectables, implants, hormonal IUDs) remain more limited in the included primary studies, and global summaries rarely distinguish their effects from those of combined oral contraceptives, underscoring the need for more granular analyses by method type [55].

#### 3.3.4. Lifestyle and Socioeconomic Factors

##### Tobacco Smoking

Tobacco smoking remains one of the most consistently established non-infectious risk factors for cervical cancer. Pooled case–control analyses demonstrate that current smokers have a significantly higher risk of high-grade CIN and invasive cervical cancer compared with never-smokers, with evidence of a dose–response relationship according to both intensity and duration of smoking [17,63]. Large multi-country cohort studies from Europe and North America have similarly shown that current smoking among HPV-positive women is associated with increased incidence of CIN3+ and invasive cancer, while former smokers generally exhibit intermediate risk levels [42,65]. Hospital- and community-based studies in Africa and Asia have also reported a higher likelihood of cervical cancer among smokers, particularly when smoking co-occurs with early sexual debut and high parity [66,67]. Global risk factor summaries classify active tobacco smoking as a causal co-factor and increasingly acknowledge exposure to second-hand smoke as a potential, albeit less well-quantified, contributor [3,57].

##### Body Mass Index, Obesity, and Other Lifestyle Factors

The evidence concerning body mass index (BMI), obesity, and other metabolic factors remains inconsistent across the included studies. Some hospital-based investigations have suggested an increased risk of cervical adenocarcinoma—and possibly squamous cell carcinoma—among women with overweight or obesity, particularly in settings where obesity is correlated with lower screening participation [61,66]. However, other analyses, after adjustment for parity, smoking, and socioeconomic indicators, have reported weak or null independent associations. In certain screened populations, overweight women appear more likely to have benign findings but are also less likely to participate regularly in screening, further complicating interpretation [5,30]. Comparative global assessments of attributable cancer burden attribute only a small proportion of cervical cancer disability-adjusted life years (DALYs) directly to metabolic risk factors such as high BMI, low physical activity, and unhealthy diet [67,68].

##### Socioeconomic Status and Education

A clear socioeconomic gradient in cervical cancer burden is evident across multiple studies. National and subnational analyses consistently show higher incidence, later-stage diagnosis, and increased mortality among women with lower educational attainment, lower income, and residence in deprived areas—even in settings with universal health coverage [5,46]. Community-based surveys in Ghana, India, and other low- and middle-income countries have demonstrated markedly lower awareness of cervical cancer, HPV, and screening services among women with little or no formal education, with correspondingly lower screening participation compared with more educated women [26,39,46,69]. In high-income countries, registry-based studies similarly indicate that women from deprived neighborhoods and minority ethnic groups are more likely to present with advanced disease and experience poorer survival, even after adjustment for stage and treatment received [28,30]. Global burden analyses have linked higher national cervical cancer rates to lower Human Development Index scores, reduced health expenditure, and weaker health system performance [2,5].

##### Place of Residence, Health Insurance, and Access-Related Factors

Geographic and health system factors profoundly influence risk through their effect on access to prevention, early detection, and treatment. Subnational analyses in the United States have shown persistently higher incidence and mortality in counties with long-standing low screening coverage—counties that are typically more rural, poorer, and have a higher proportion of uninsured women or those covered only by public insurance [4]. Community-based studies in Ghana, Uganda, and other African settings report lower screening participation and higher prevalence of high-grade lesions among rural residents and those living in informal urban settlements, reflecting greater distance to services, fewer facilities, and persistent financial and cultural barriers [26,69,70]. In high-income countries, lack of insurance and certain disabilities are associated with lower guideline-concordant screening and higher probability of advanced-stage diagnosis [28,71]. Collectively, these findings indicate that place of residence and access to health services constitute major structural determinants of lifetime risk of cervical cancer incidence and mortality.

#### 3.3.5. Host, Genetic, and Immunological Factors

##### Genetic Susceptibility Variants and Polymorphisms

Several genome-wide association studies (GWAS) and candidate-gene analyses included in this review have identified host genetic variants associated with susceptibility to cervical neoplasia. Large international GWAS conducted among women of European and Asian ancestry have highlighted loci in the HLA region as well as other immune-related genes, with some variants linked to increased risk and others to protection against high-grade CIN and invasive cancer [21,22,23]. Additional analyses have identified single-nucleotide polymorphisms (SNPs) associated with seropositivity to HPV16 and other carcinogenic types, suggesting that genetic factors may influence both initial infection susceptibility and subsequent disease progression [23,72]. Mendelian randomization studies using genetically proxied traits have further suggested possible causal effects of genetically influenced smoking propensity and parity on cervical cancer risk [24]. Overall, these findings support a role for host genetic susceptibility—particularly in antigen presentation and immune regulation pathways—although the variants identified to date explain only a relatively small proportion of overall risk.

##### Comorbidities and Non-Communicable Diseases

Evidence regarding non-communicable comorbidities as independent risk factors remains relatively limited. However, several clinical cohort studies indicate that women with hypertension, diabetes, or multiple chronic conditions tend to present with more advanced cervical cancer and experience poorer survival, even after adjustment for age and stage at diagnosis [30,54]. These associations appear to be driven, at least in part, by later presentation and reduced tolerance of intensive cancer treatment. Comparative global burden assessments attribute only a modest proportion of cervical cancer DALYs directly to metabolic risk factors [68,73]. Thus, the available evidence suggests that comorbidities play a more prominent role in prognosis and treatment outcomes than in primary disease causation.

##### Other Immunological and Host Conditions

Beyond HIV-related immunosuppression and inherited genetic variants, relatively few primary studies in this review directly examined other immunological states or host conditions in relation to cervical carcinogenesis. Global risk factor summaries note that chronic immunosuppressive conditions (e.g., solid organ transplantation, long-term use of immunosuppressive drugs) are likely to increase the risk of persistent HPV infection and progression, consistent with patterns observed in other HPV-related cancers [3,55]. However, quantitative data on these specific populations were not prominent among the included primary studies. Overall, alterations in immune function are recognized as important co-determinants of risk, though their contribution—outside of HIV and hereditary susceptibility—remains incompletely characterized in contemporary epidemiological literature.

### 3.4. Patterns of Screening and Prevention

#### 3.4.1. Types of Screening Tests Employed

Evidence from global reports and primary studies indicates that a range of screening modalities is currently in use, with the choice of method largely determined by regional resources and health system capacity. In many high-income countries and some upper-middle-income settings with established programs, cytology-based screening (conventional Pap smear or liquid-based cytology) remains a mainstay. In these contexts, HPV DNA testing is frequently used as a co-test or for triage purposes [8,45,63].

Primary HPV screening has gained increasing traction in recent years, particularly in high-income countries and in pilot or early scale-up phases in selected middle-income settings, sometimes incorporating self-sampling [46,74]. In low-resource environments, visual inspection with acetic acid (VIA) and other low-cost approaches continue to be widely implemented, often within a “screen-and-treat” paradigm that enables same-day treatment of screen-positive women using cryotherapy or thermal ablation [26,61].

Comparative performance studies consistently report that HPV-based strategies exhibit higher sensitivity for the detection of CIN2+ than either conventional cytology or VIA, supporting the global strategic shift toward primary HPV screening [8,45].

#### 3.4.2. Coverage, Participation, and Determinants of Screening Uptake

Despite the availability of effective screening tests, coverage remains highly variable both between and within countries. Global reports indicate that many high-income countries have achieved moderate-to-high participation through organized programs, whereas in most low- and middle-income countries coverage is low and screening is predominantly opportunistic [3,46].

Subnational analyses in high-income settings reveal persistent disparities, with lower participation among women residing in deprived, rural, or minority communities, particularly where active invitation systems are absent or primary care access is limited [4,75]. Community-based surveys in Ghana, Uganda, India, and Bangladesh show that only a small minority of women have ever undergone screening, with key barriers including limited awareness of Pap smear or HPV testing, perceived low personal risk, direct and indirect costs, distance to services, and sociocultural norms [26,39,40,69].

Healthcare provider recommendation emerges as one of the strongest predictors of screening uptake across studies, while negative provider attitudes, inadequate training, or competing workload priorities are frequently cited as programmatic barriers [70,76]. Programmatic evaluations from several high-income and selected middle-income countries demonstrate that organized, population-based screening programs with centralized invitation systems and quality assurance achieve substantially higher and more equitable coverage than opportunistic models [45,46,48]. In addition, HPV self-sampling strategies have consistently improved participation among under-screened populations, including rural residents, socioeconomically disadvantaged women, and those facing cultural or logistical barriers, without compromising test sensitivity [26,39,74]. Despite their strong potential to expand coverage, the real-world scalability of HPV DNA testing and self-sampling in low-resource settings remains constrained by laboratory capacity, specimen transport, result turnaround time, and linkage-to-care systems for positive cases [26,46,74]. Integration with decentralized testing platforms, digital tracking, and community-based follow-up mechanisms is therefore critical to translate screening access into timely diagnosis and treatment. In parallel, emerging evidence supporting single-dose HPV vaccination offers an important opportunity to improve feasibility, affordability, and equity in high-burden settings, particularly where multi-dose delivery and completion remain challenging.

#### 3.4.3. HPV Vaccination (Policy and Programmatic Level)

Policy documents and epidemiological reports document the rapid expansion of HPV vaccination programs over the past 15–20 years, albeit with marked global inequities. The WHO Global Strategy to Accelerate the Elimination of Cervical Cancer as a Public Health Problem sets ambitious targets for HPV vaccination, screening, and treatment coverage, defining elimination as achieving an age-standardized incidence rate below a specified threshold [11].

By the early 2020s, most high-income countries and a growing number of middle-income countries had introduced routine HPV vaccination for girls (and in some cases boys), frequently through school-based delivery platforms. However, completion rates remain substantially lower in many low-income countries and among marginalized populations in wealthier settings [2,3].

Global and national syntheses emphasize that high-coverage HPV vaccination, when combined with high-coverage screening and timely treatment of precancerous lesions, can dramatically reduce incidence and move most countries toward elimination. Achieving these gains requires sustained financing, health system strengthening, and deliberate efforts to address social and geographic inequities in service access [46,55]. The WHO 70–90–70 targets aim to achieve 90% HPV vaccination coverage among girls by age 15, 70% screening coverage at least twice in a lifetime, and 90% treatment of screen-detected lesions, providing a unified global framework for elimination [11,13]. In settings with sustained high vaccination coverage, modeling studies suggest that screening intervals may be safely extended and primary HPV-based strategies prioritized, although continued screening remains necessary for several decades due to cohort effects [7,13,45].

#### 3.4.4. Adjuvant HPV Vaccination After Diagnostic or Excisional Treatment

Emerging evidence supports the role of HPV vaccination as an adjuvant strategy following diagnostic evaluation or therapeutic procedures such as colposcopy, loop electrosurgical excision procedure (LEEP), or cervical conization. A recent large-scale population-based study demonstrated that post-treatment HPV vaccination was associated with a significant reduction in the risk of recurrent high-grade cervical intraepithelial neoplasia and subsequent HPV-related disease [77].

Given that persistent high-risk HPV infection is a necessary cause of cervical carcinogenesis and that viral persistence and immune clearance critically influence progression and recurrence [14,55,63], post-treatment vaccination may enhance immune-mediated viral control and reduce reinfection or persistence after excisional treatment.

From a population perspective, integration of vaccination with screening and timely treatment has been shown to accelerate progress toward cervical cancer elimination in modeling studies and real-world programs [7,13,45]. Extending vaccination strategies to the post-treatment setting may therefore further strengthen the effectiveness and equity of cervical cancer prevention pathways, particularly among women at elevated risk of recurrence.

## 4. Discussion

This narrative synthesis of primary epidemiological studies, global burden estimates, and existing reviews confirms that cervical cancer remains a major but highly inequitable cause of cancer incidence and mortality worldwide. The burden falls disproportionately on low- and lower-middle-income countries—particularly in sub-Saharan Africa, parts of South and Southeast Asia—as well as on socioeconomically disadvantaged and geographically marginalized groups within higher-income settings [5,12]. Within-country analyses further demonstrate clear gradients by socioeconomic status, rurality, and health system performance, with the highest rates observed in underserved rural and deprived urban populations [4,46].

Temporal trends reveal important improvements in many high-income and upper-middle-income countries, where long-established cytology-based screening and earlier HPV vaccination introduction have driven substantial declines in age-standardized incidence and mortality. In contrast, rates remain high, stable, or even rising in several low-resource regions. Collectively, these patterns indicate that cervical cancer is increasingly concentrated among women who are socially and geographically marginalized, and that progress toward elimination will depend critically on closing persistent gaps in prevention and care.

The observed geographic and temporal patterns align closely with recent global assessments and narrative reviews. GLOBOCAN and GBD analyses repeatedly show cervical cancer as the fourth most common cancer among women globally, yet the first or second leading cause of cancer death in many low Human Development Index countries, where over 80% of deaths occur [1,2]. Recent updates confirm that the largest disparities—exceeding tenfold differences in age-standardized rates—persist between highest-burden (eastern/southern Africa) and lowest-burden (North America, Western Europe, parts of West Asia) regions [46,78,79].

The central role of persistent high-risk HPV infection is strongly reaffirmed, with HPV16/18 responsible for the majority of cases globally and other carcinogenic types showing important regional variation [14,15]. The marked excess risk among women living with HIV, and the concentration of HIV-associated cases in eastern and southern Africa, are consistent with pooled analyses demonstrating several-fold increases in incidence among HIV-positive women [19,79].

Sexual and reproductive factors (early sexual debut, multiple partners, high parity, early first birth) show the expected associations, in line with large pooled case–control studies and prior reviews [17,43,62]. Long-term oral contraceptive use is associated with a modest increase in risk among HPV-positive women, consistent with earlier pooled analyses [18,41].

Tobacco smoking emerges as the most robust non-infectious co-factor, with consistent dose–response relationships reported across observational and genetically informed studies [63,65]. In contrast, associations with BMI and other metabolic factors remain inconsistent and generally weak after appropriate adjustment, aligning with global burden estimates that attribute only a small fraction of cervical cancer DALYs to these exposures [67,68].

Socioeconomic position, education, rural residence, and health insurance status show strong and persistent associations with higher incidence, later-stage diagnosis, and poorer survival—patterns that reflect structural barriers at every stage of the care continuum [9,29]. The transition toward primary HPV screening, the promise of self-sampling, and the uneven but ongoing global expansion of HPV vaccination are consistent with international policy documents and modeling studies [11,13].

Genetic susceptibility (particularly HLA-region variants) and comorbidities play more limited but non-negligible roles, predominantly in disease progression and prognosis rather than primary causation [21,23,30].

Limitations of the evidence base include persistent geographic data gaps, reliance on cross-sectional or hospital-based samples in many settings, heterogeneity in exposure measurement, and incomplete linkage between screening/vaccination data and disease outcomes. Despite these constraints, the overall coherence of findings across diverse study designs and settings lends confidence to the major conclusions.

The synthesis highlights clear priorities: scaling high-quality HPV vaccination and screening in high-burden, low-coverage populations; integrating services into primary care, sexual/reproductive health, and HIV platforms; and systematically addressing structural barriers (poverty, gender norms, stigma, transport, weak information systems). Beyond summarizing established associations, this review advances prior global narrative reviews by integrating epidemiological burden, multilevel risk factors, prevention strategies, and equity considerations, and by explicitly addressing the gap between national elimination targets and persistent subnational inequities. Several specific research gaps emerge: (1) longitudinal cohort studies in high-burden settings to better characterize causal pathways and life-course exposures; (2) pragmatic implementation trials evaluating scalable delivery models for HPV testing, self-sampling, and treatment linkage; and (3) equity-focused evaluations assessing differential reach and effectiveness across socioeconomic and geographic strata.

## 5. Conclusions

This narrative review integrates primary epidemiological studies, global burden estimates, and targeted reviews to present a comprehensive picture of the current epidemiology of cervical cancer. The disease remains a major yet profoundly inequitable cause of cancer morbidity and mortality, with the greatest burden borne by low- and lower-middle-income countries and by socioeconomically and geographically disadvantaged groups within higher-income settings.

Persistent infection with high-risk HPV types is reconfirmed as the necessary cause of virtually all cases, while progression to invasive disease results from a complex interplay of sexual/reproductive history, hormonal exposures, tobacco use, HIV-related and other immunosuppression, socioeconomic position, and health system performance. Striking disparities in incidence, stage at diagnosis, and survival reflect both biological and deeply structural determinants.

At the same time, the evidence base demonstrates substantial opportunities for prevention. High-quality screening and HPV vaccination have already contributed to meaningful reductions in several high-income and transitional settings, and performance data strongly support the continued shift toward primary HPV-based screening. However, coverage of both screening and vaccination remains low and inequitable in many of the highest-risk populations, constrained by resource limitations, health system weaknesses, and enduring social, geographic, and cultural barriers.

Progress toward elimination depends not only on the availability of effective technologies but on their equitable, accessible, trusted, and integrated delivery—with particular attention to rural, poor, and marginalized women and to regions with high HIV prevalence. Filling persistent data gaps through strengthened registration systems, longitudinal cohorts, and implementation research will be essential to refine burden estimates, clarify co-factor roles, and guide the design and evaluation of equitable elimination strategies.

## Figures and Tables

**Table 1:** Factors associated with cervical cancer risk, progression, and prevention.

Domain	Factor/Determinant	Protective/ Preventive	Predisposing/ Risk-Enhancing	Inconsistent/ Insufficient Evidence
Infection-related factors	Persistent high-risk HPV infection (necessary cause)		✓	
	Co-infection with other sexually transmitted infections (non-HPV STIs)			✓
	HIV infection/HIV-associated immunosuppression		✓	
Sexual and reproductive behaviors	Early age at first sexual intercourse/early age at marriage		✓	
	Multiple lifetime sexual partners		✓	
	Male partner with multiple partners or STI history		✓	
	High parity (multiple full-term pregnancies; dose–response reported)		✓	
	Early age at first full-term birth (risk amplifier among HPV-infected women)		✓	
Hormonal factors	Ever-use of combined oral contraceptives (COCs) (modest risk increase)		✓	
	Longer duration of COC use (particularly >5 years)		✓	
	Non-oral hormonal methods (injectables/implants/hormonal IUDs)			✓
Lifestyle and metabolic factors	Active tobacco smoking (dose–response; robust co-factor)		✓	
	Second-hand smoke exposure			✓
	Body mass index (BMI), overweight/obesity and related metabolic factors			✓
	Low physical activity/unhealthy diet (small attributable fraction in burden assessments)			✓
Socioeconomic and access-related determinants	Lower socioeconomic status/lower educational attainment		✓	
	Residence in deprived neighborhoods/minority or marginalized groups		✓	
	Rural residence/geographic barriers		✓	
	Lack of health insurance and certain disabilities (lower screening; later diagnosis)		✓	
	Low or uneven screening coverage (opportunistic vs. organized)		✓	
Host, genetic, and immunological factors	Genetic susceptibility (e.g., HLA-region and immune-related variants; modest effects)		✓	
	Other immunosuppressive conditions (e.g., transplantation; long-term immunosuppressive drugs)			✓
Comorbidities and prognosis-related factors	Hypertension, diabetes, and multimorbidity (linked mainly to advanced presentation and poorer survival)		✓	
Screening and prevention (programmatic)	High-coverage HPV vaccination (especially when combined with screening and timely treatment)	✓		
	High-coverage screening and timely treatment of precancerous lesions	✓		
	Healthcare provider recommendation (strong predictor of screening uptake)	✓		

## Data Availability

No new data were created or analyzed in this study. Data sharing is not applicable to this article.

## References

[1] Bray F., Ferlay J., Soerjomataram I., Siegel R.L., Torre L.A., Jemal A. (2018). Global cancer statistics 2018: GLOBOCAN estimates of incidence and mortality worldwide for 36 cancers in 185 countries. CA Cancer J. Clin..

[2] Momenimovahed Z., Mazidimoradi A., Maroofi P., Allahqoli L., Salehiniya H., Alkatout I. (2023). Global, regional and national burden, incidence, and mortality of cervical cancer. Cancer Rep..

[3] World Health Organization Cervical Cancer 2024. https://www.who.int/news-room/fact-sheets/detail/cervical-cancer.

[4] Amboree T.L., Montealegre J.R., Damgacioglu H., Orr B., Wrangle J., Sonawane K., Deshmukh A.A. (2025). County-Level Cervical Cancer Screening Coverage and Differences in Incidence and Mortality. JAMA Netw. Open.

[5] Huang J., Deng Y., Boakye D., Tin M.S., Lok V., Zhang L., Lucero-Prisno D.E., Xu W., Zheng Z.J., Elcarte E. (2022). Global distribution, risk factors, and recent trends for cervical cancer: A worldwide country-level analysis. Gynecol. Oncol..

[6] Piñeros M., Saraiya M., Baussano I., Bonjour M., Chao A., Bray F. (2021). The role and utility of population-based cancer registries in cervical cancer surveillance and control. Prev. Med..

[7] Brisson M., Kim J.J., Canfell K., Drolet M., Gingras G., Burger E.A., Martin D., Simms K.T., Bénard É., Boily M.-C. (2020). Impact of HPV vaccination and cervical screening on cervical cancer elimination: A comparative modelling analysis in 78 low-income and lower-middle-income countries. Lancet.

[8] Cancer IAfRo (2022). Global Cancer Observatory: Cancer Today (GLOBOCAN 2022).

[9] Buskwofie A., David-West G., Clare C.A. (2020). A Review of Cervical Cancer: Incidence and Disparities. J. Natl. Med. Assoc..

[10] Pilleron S., Soto-Perez-de-Celis E., Vignat J., Ferlay J., Soerjomataram I., Bray F., Sarfati D. (2021). Estimated global cancer incidence in the oldest adults in 2018 and projections to 2050. Int. J. Cancer.

[11] World Health Organization (2020). Global Strategy to Accelerate the Elimination of Cervical Cancer as a Public Health Problem.

[12] Bray F., Laversanne M., Sung H., Ferlay J., Siegel R.L., Soerjomataram I., Jemal A. (2024). Global cancer statistics 2022: GLOBOCAN estimates of incidence and mortality worldwide for 36 cancers in 185 countries. CA Cancer J. Clin..

[13] Canfell K., Kim J.J., Brisson M., Keane A., Simms K.T., Caruana M., Burger E.A., Martin D., Nguyen D.T.N., Bénard É. (2020). Mortality impact of achieving WHO cervical cancer elimination targets: A comparative modelling analysis in 78 low-income and lower-middle-income countries. Lancet.

[14] Walboomers J.M., Jacobs M.V., Manos M.M., Bosch F.X., Kummer J.A., Shah K.V., Snijders P.J., Peto J., Meijer C.J., Muñoz N. (1999). Human papillomavirus is a necessary cause of invasive cervical cancer worldwide. J. Pathol..

[15] Dong B., Lu Z., Yang T., Wang J., Zhang Y., Tuo X., Wang J., Lin S., Cai H., Cheng H. (2025). Development, validation, and clinical application of a machine learning model for risk stratification and management of cervical cancer screening based on full-genotyping hrHPV test (SMART-HPV): A modelling study. Lancet Reg. Health West. Pac..

[16] Arbyn M., Weiderpass E., Bruni L., de Sanjosé S., Saraiya M., Ferlay J., Bray F. (2020). Estimates of incidence and mortality of cervical cancer in 2018: A worldwide analysis. Lancet Glob. Health.

[17] Plummer M., Herrero R., Franceschi S., Meijer C.J., Snijders P., Bosch F.X., de Sanjosé S., Muñoz N. (2003). Smoking and cervical cancer: Pooled analysis of the IARC multi-centric case--control study. Cancer Causes Control.

[18] Castellsagué X., Muñoz N. (2003). Chapter 3: Cofactors in human papillomavirus carcinogenesis--role of parity, oral contraceptives, and tobacco smoking. J. Natl. Cancer Inst. Monogr..

[19] Lin S., Gao K., Gu S., You L., Qian S., Tang M., Wang J., Chen K., Jin M. (2021). Worldwide trends in cervical cancer incidence and mortality, with predictions for the next 15 years. Cancer.

[20] Hakim R.U., Amin T., Ul Islam S.M.B. (2025). Advances and Challenges in Cervical Cancer: From Molecular Mechanisms and Global Epidemiology to Innovative Therapies and Prevention Strategies. Cancer Control.

[21] Chen D., Juko-Pecirep I., Hammer J., Ivansson E., Enroth S., Gustavsson I., Feuk L., Magnusson P.K., McKay J.D., Wilander E. (2013). Genome-wide association study of susceptibility loci for cervical cancer. J. Natl. Cancer Inst..

[22] Shi Y., Li L., Hu Z., Li S., Wang S., Liu J., Wu C., He L., Zhou J., Li Z. (2013). A genome-wide association study identifies two new cervical cancer susceptibility loci at 4q12 and 17q12. Nat. Genet..

[23] Beckhaus T., Kachuri L., Nakase T., Schürmann P., Eisenblätter R., Geerts M., Böhmer G., Strauß H.G., Hirchenhain C., Schmidmayr M. (2025). Genome-Wide Association Analyses of HPV16 and HPV18 Seropositivity Identify Susceptibility Loci for Cervical Cancer. J. Med. Virol..

[24] Wang F., Liu Y., Jia J., Bai X., Zhang M., Ye X., Wang L., Bai Y. (2025). Causality between 22 personal traits and cervical cancer: A two-sample Mendelian randomization study. J. Affect. Disord..

[25] Shrestha A.D., Neupane D., Vedsted P., Kallestrup P. (2018). Cervical Cancer Prevalence, Incidence and Mortality in Low and Middle Income Countries: A Systematic Review. Asian Pac. J. Cancer Prev..

[26] Adzigbli L.A., Aboagye R.G., Adeleye K., Osborne A., Ahinkorah B.O. (2025). Cervical cancer screening uptake and its predictors among women aged 30–49 in Ghana: Providing evidence to support the World Health Organization’s cervical cancer elimination initiative. BMC Infect. Dis..

[27] Emagneneh T., Mulugeta C., Ejigu B., Alamrew A., Hiwot A.Y., Feleke S.F. (2025). Survival status of women with cervical cancer in Sub-Saharan Africa: A systematic review and meta-analysis, 2024. Front. Oncol..

[28] Rauh-Hain J.A., Melamed A., Schaps D., Bregar A.J., Spencer R., Schorge J.O., Rice L.W., Del Carmen M.G. (2018). Racial and ethnic disparities over time in the treatment and mortality of women with gynecological malignancies. Gynecol. Oncol..

[29] Ezeamii J.C., Edwards Q., Omale J., Ezeamii P.C., Idoko B., Ejembi E.V. (2024). Risk beyond the pap: A review of key epidemiological studies on cervical cancer risk factors and populations at highest risk. World J. Adv. Res. Rev..

[30] Balasubramaniam G., Gaidhani R., Khan A., Saoba S., Mahantshetty U., Maheshwari A. (2020). Survival rate of cervical cancer from a study conducted in India. Indian J. Med. Sci..

[31] Hamid M.K.I., Hasneen S., Lima A.K., Shawon S.R., Shahriar M., Anjum R. (2025). Cervical cancer trends, HPV vaccine utilization, and screening in low- and lower-middle-income countries: An updated review. Ther. Adv. Vaccines Immunother..

[32] Zhang S., Xu H., Zhang L., Qiao Y. (2020). Cervical cancer: Epidemiology, risk factors and screening. Chin. J. Cancer Res..

[33] Brotherton J.M.L., Vajdic C.M., Nightingale C. (2025). The socioeconomic burden of cervical cancer and its implications for strategies required to achieve the WHO elimination targets. Expert Rev. Pharmacoecon. Outcomes Res..

[34] Win T.Z.K., Simms K., Thinn M.M., Tin K.N., Aung S., Feletto E., Bateson D., Canfell K. (2025). A Review of Human Papillomavirus Prevalence and Cervical Cancer in Myanmar. J. Epidemiol. Glob. Health.

[35] Vali M., Maleki Z., Nikbakht H.-A., Hassanipour S., Kouhi A., Nazemi S., Hajizade-valokolaee M., Nayeb M., Ghaem H. (2023). Survival rate of cervical cancer in Asian countries: A systematic review and meta-analysis. BMC Women’s Health.

[36] Yao Y., Zhu Q., Li X., Sun K., Zheng R. (2025). Global patterns of cervical cancer incidence and mortality: Updated statistics and an overview of temporal trends from 2003 to 2017. Med. Plus.

[37] Neumeyer S., Tanaka L.F., Liang L.A., Klug S.J. (2023). Epidemiology of cervical cancer in elderly women: Analysis of incidence, treatment, and survival using German registry data. Cancer Med..

[38] Semenov V.V., Kriachkova L.V., Boată A.E., Geantă M.M. (2025). Trends in cervical cancer epidemiology and vaccination coverage in Ukraine. Wiad. Lek..

[39] Bose S., Mandal R., Banerjee D., Vernekar M., Siddiqi M., Chakrabarti J., Sankaranarayanan R., Lucas E., Muwonge R., Basu P. (2025). Analysis of time trends of prevalence of high-risk HPV infections, high grade cervical precancer and cervical cancer disease in women from Eastern India over 20 years—Pooled analysis from three studies. Cancer Epidemiol..

[40] Islam A.I., Ahammed E., Nisa N.A., Mim A.A., Akhter F.B., Amin F. (2025). Knowledge, Attitudes, Practices, and Risk Factors Related to Breast and Cervical Cancer Among Female Medical Students in Comilla, Bangladesh. Asia Pac. J. Surg. Adv..

[41] Moreno V., Bosch F.X., Muñoz N., Meijer C.J., Shah K.V., Walboomers J.M., Herrero R., Franceschi S. (2002). Effect of oral contraceptives on risk of cervical cancer in women with human papillomavirus infection: The IARC multicentric case-control study. Lancet.

[42] Roura E., Castellsagué X., Pawlita M., Travier N., Waterboer T., Margall N., Bosch F.X., de Sanjosé S., Dillner J., Gram I.T. (2014). Smoking as a major risk factor for cervical cancer and pre-cancer: Results from the EPIC cohort. Int. J. Cancer.

[43] Roura E., Travier N., Waterboer T., de Sanjosé S., Bosch F.X., Pawlita M., Pala V., Weiderpass E., Margall N., Dillner J. (2016). The Influence of Hormonal Factors on the Risk of Developing Cervical Cancer and Pre-Cancer: Results from the EPIC Cohort. PLoS ONE.

[44] Wang H., Nie K., Liu Z., Zhao Y., Ha Y., Zhang H., Mao D. (2024). Analysis of risk factors associated with cervical HPV infection and their effects on female sexual function and anxiety: A multicenter cross-sectional study based on Chinese women. Front. Oncol..

[45] Luu X.Q., Jun J.K., Suh M., Oh J.-K., Yu S.-Y., Choi K.S. (2025). Cervical Cancer Screening, HPV Vaccination, and Cervical Cancer Elimination. JAMA Netw. Open.

[46] Ma S., Sun K., Han B., Zheng R., Wei W. (2025). Cervical cancer burden and trends in China, 2000-2020: Asia-Pacific international comparisons and insights for elimination goals. Cancer Biol. Med..

[47] Yang M., Du J., Lu H., Xiang F., Mei H., Xiao H. (2022). Global trends and age-specific incidence and mortality of cervical cancer from 1990 to 2019: An international comparative study based on the Global Burden of Disease. BMJ Open.

[48] Wang M., Jin Y., Zheng Z.J. (2023). The association of cervical cancer screening and quality of care: A systematic analysis of the Global Burden of Disease Study 2019. J. Glob. Health.

[49] Zhang M., Chen J., Cui M., Jia J., Zhao M., Zhou D., Zhu L., Luo L. (2024). Analysis of the global burden of cervical cancer in young women aged 15–44 years old. Eur. J. Public Health.

[50] Stuebs F.A., Beckmann M.W., Pöschke P., Heindl F., Emons J., Gaß P., Häberle L. (2025). The Epidemiology of Cervical Cancer in Germany: A Registry-Based Analysis of Incidence, Survival, and Tumor Characteristics (2003–2021). Dtsch. Arztebl. Int..

[51] Seweryn M., Leszczyńska A., Jakubowicz J., Banaś T. (2025). Cervical cancer in Poland—Epidemiology, prevention, and treatment pathways. Oncol. Clin. Pract..

[52] Jedy-Agba E., Joko W.Y., Liu B., Buziba N.G., Borok M., Korir A., Masamba L., Manraj S.S., Finesse A., Wabinga H. (2020). Trends in cervical cancer incidence in sub-Saharan Africa. Br. J. Cancer.

[53] Sengayi-Muchengeti M., Joko-Fru W.Y., Miranda-Filho A., Egue M., Akele-Akpo M.T., N’da G., Mathewos A., Buziba N., Korir A., Manraj S. (2020). Cervical cancer survival in sub-Saharan Africa by age, stage at diagnosis and Human Development Index: A population-based registry study. Int. J. Cancer.

[54] Liu G., Yang Z., Wang D. (2023). A Bayesian network predicting survival of cervical cancer patients-Based on surveillance, epidemiology, and end results. Cancer Sci..

[55] National Cancer Institute Cervical Cancer Causes, Risk Factors, and Prevention 2024. https://www.cancer.gov/types/cervical/causes-risk-prevention.

[56] Sucato A., Serra N., Buttà M., Gregorio L.D., Pistoia D., Capra G. (2025). Human Papillomavirus Infection in Partners of Women Attending Cervical Cancer Screening: A Pilot Study on Prevalence, Distribution, and Potential Use of Vaccines. Vaccines.

[57] American Cancer Society (2025). Cervical Cancer Risk Factors. https://www.cancer.org/cancer/types/cervical-cancer/causes-risks-prevention/risk-factors.html.

[58] UKCR (2023). Cervical Cancer Risk. https://www.cancerresearchuk.org/health-professional/cancer-statistics/statistics-by-cancer-type/cervical-cancer/risk-factors.

[59] Zohra F.-T., Islam R.Z., Tasnim F., Howlader B., Md S., Parveen K. (2025). Risk Factors, Health-Seeking Behavior, Attitudes, and Knowledge Regarding Cervical Carcinoma Among Rural Women in Bangladesh. Asia Pac. J. Surg. Adv..

[60] Nadarzynski T., Waller J., Robb K.A., Marlow L.A. (2012). Perceived risk of cervical cancer among pre-screening age women (18–24 years): The impact of information about cervical cancer risk factors and the causal role of HPV. Sex. Transm. Infect..

[61] Taye B.T., Mihret M.S., Muche H.A. (2021). Risk factors of precancerous cervical lesions: The role of women’s socio-demographic, sexual behavior and body mass index in Amhara region referral hospitals; case-control study. PLoS ONE.

[62] Appleby P., Beral V., Berrington de González A., Colin D., Franceschi S., Goodhill A., Green J., Peto J., Plummer M., Sweetland S. (2007). Cervical cancer and hormonal contraceptives: Collaborative reanalysis of individual data for 16,573 women with cervical cancer and 35,509 women without cervical cancer from 24 epidemiological studies. Lancet.

[63] Luhn P., Walker J., Schiffman M., Zuna R.E., Dunn S.T., Gold M.A., Smith K., Mathews C., Allen R.A., Zhang R. (2013). The role of co-factors in the progression from human papillomavirus infection to cervical cancer. Gynecol. Oncol..

[64] Guo C., Zhan B., Li M.Y., Yue L., Zhang C. (2024). Association between oral contraceptives and cervical cancer: A retrospective case-control study based on the National Health and Nutrition Examination Survey. Front. Pharmacol..

[65] Su L., Xu R., Ren Y., Meng C., Wang P., Wu Q., Song L., Du Z. (2025). Smoking exposure and cervical cancer risk: Integrating observational and genetic evidence. J. Int. Med. Res..

[66] Olajide N., Robb K., Niedzwiedz C., Jani B. (2022). Cervical cancer risk factors in eight west African countries: Cross-sectional analysis of the demographic and health survey 2017–20. Lancet.

[67] Kim J.Y., Lee D.W., Kim M.J., Shin J.E., Shin Y.J., Lee H.N. (2021). Secondhand smoke exposure, diabetes, and high BMI are risk factors for uterine cervical cancer: A cross-sectional study from the Korea national health and nutrition examination survey (2010–2018). BMC Cancer.

[68] Cheng L.Y., Zhao J.Q., Zou T.T., Xu Z.H., Lv Y. (2025). Cervical cancer burden and attributable risk factors across different age and regions from 1990 to 2021 and future burden prediction: Results from the global burden of disease study 2021. Front. Oncol..

[69] Mwaka A.D., Orach C.G., Were E.M., Lyratzopoulos G., Wabinga H., Roland M. (2016). Awareness of cervical cancer risk factors and symptoms: Cross-sectional community survey in post-conflict northern Uganda. Health Expect..

[70] Oringtho S., Mwaka A.D., Garimoi Orach C., Wabinga H. (2024). Awareness of cervical cancer risk factors and preventive approaches, and perceived causes of cervical cancer among secondary school girls: A cross-sectional study in Northern Uganda. Ann. Med..

[71] Orji A.F., Gimm G., Desai A., Parekh T. (2024). The Association of Cervical Cancer Screening With Disability Type Among U.S. Women (Aged 25–64 Years). Am. J. Prev. Med..

[72] Leo P.J., Madeleine M.M., Wang S., Schwartz S.M., Newell F., Pettersson-Kymmer U., Hemminki K., Hallmans G., Tiews S., Steinberg W. (2017). Defining the genetic susceptibility to cervical neoplasia-A genome-wide association study. PLoS Genet..

[73] Wu S., Jiao J., Yue X., Wang Y. (2024). Cervical cancer incidence, mortality, and burden in China: A time-trend analysis and comparison with England and India based on the global burden of disease study 2019. Front. Public Health.

[74] Yang D., Zhang J., Cui X., Ma J., Wang C., Piao H. (2022). Risk Factors Associated With Human Papillomavirus Infection, Cervical Cancer, and Precancerous Lesions in Large-Scale Population Screening. Front. Microbiol..

[75] Bowden S.J., Bodinier B., Kalliala I., Zuber V., Vuckovic D., Doulgeraki T., Whitaker M.D., Wielscher M., Cartwright R., Tsilidis K.K. (2021). Genetic variation in cervical preinvasive and invasive disease: A genome-wide association study. Lancet Oncol..

[76] Higashi R.T., Tiro J.A., Winer R.L., Ornelas I.J., Bravo P., Quirk L., Kessler L.G. (2023). Understanding the effect of new U.S. cervical cancer screening guidelines and modalities on patients’ comprehension and reporting of their cervical cancer screening behavior. Prev. Med. Rep..

[77] Han J., Zhang L., Chen Y., Zhang Y., Wang L., Cai R., Zhu L. (2025). Global HPV vaccination programs and coverage rates: A systematic review. EClinicalMedicine.

[78] Society A.C. (2024). Global Cancer Facts & Figures.

[79] Stelzle D., Tanaka L.F., Lee K.K., Ibrahim Khalil A., Baussano I., Shah A.S.V., McAllister D.A., Gottlieb S.L., Klug S.J., Winkler A.S. (2021). Estimates of the global burden of cervical cancer associated with HIV. Lancet Glob. Health.

